# Puerarin: A Potential Therapeutic for SARS-CoV-2 and Hantavirus Co-Infection

**DOI:** 10.3389/fimmu.2022.892350

**Published:** 2022-05-19

**Authors:** Weizheng Liang, Xiushen Li, Hao Wang, Kechao Nie, Qingxue Meng, Junli He, Chunfu Zheng

**Affiliations:** ^1^Central Laboratory, The First Affiliated Hospital of Hebei North University, Zhangjiakou, China; ^2^Department of Immunology, School of Basic Medical Sciences, Fujian Medical University, Fuzhou, China; ^3^Department of Obstetrics and Gynecology, Shenzhen University General Hospital, Shenzhen, China; ^4^Guangdong Key Laboratory for Biomedical Measurements and Ultrasound Imaging, School of Biomedical Engineering, Shenzhen University Health Science Center, Shenzhen, China; ^5^Shenzhen Key Laboratory, Shenzhen University General Hospital, Shenzhen, China; ^6^Department of Integrated Traditional Chinese & Western Internal Medicine, the Second Xiangya Hospital, Central South University, Changsha, China; ^7^Department of Pediatrics, Shenzhen University General Hospital Shenzhen, Guangdong, China; ^8^Department of Microbiology, Immunology and Infectious Diseases, University of Calgary, Calgary, AB, Canada

**Keywords:** bioinformatics, puerarin, SARS-CoV-2, Hantavirus, co-infection, virus immunity

## Abstract

Patients with Hantavirus-caused epidemic hemorrhagic fever (EHF) are at risk of contracting severe acute respiratory syndrome coronavirus 2 (SARS-CoV-2). However, there is currently no validated EHF/SARS-CoV-2 strategy. Several studies have recently shown Puerarin, a natural product, has potent antiviral properties. The goal of present study was to determine the mechanism of puerarin in patients with EHF/COVID-19. We use network pharmacology and bioinformatics to investigate the possible pharmacological targets, bioactivities, and molecular mechanisms of puerarin in the treatment of patients with EHF/SARS-CoV-2. The study investigated the pathogenesis of COVID-19 and EHF and the signaling pathway impacted by puerarin. 68 common genes linked to puerarin and EHF/SARS-CoV-2 were discovered during the investigation. By using protein-protein interaction (PPI) network, we identified RELA, JUN, NF-B1, NF-B2, and FOS as potential therapeutic targets. The bioactivity and signaling pathways of puerarin have also been demonstrated in the treatment of EHF and COVID-19. According to present study, puerarin could reduce excessive immune responses and inflammation through the NF-B, TNF, and HIF-1 signaling pathways. This study explored the potential therapeutic targets and mechanisms of Puerarin in the treatment of EHF/COVID-19.

## Introduction

COVID-19 is a novel zoonotic disease that causes severe flu-like symptoms and respiratory distress (1–3). The novel coronavirus causes COVID-19 termed severe acute respiratory syndrome coronavirus 2 (SARS-CoV-2), similar to the coronavirus-induced SARS (4). Since the discovery of SARS-CoV-2 in early 2020, incalculable social and economic damage has been caused (5). COVID-19 is thought to be spread through aerosols and/or droplets. Since more than 97 percent of these droplets are smaller than 50 μm, the virus spreads more easily and is more difficult to prevent (6, 7). After an incubation period of 4–14 days, most infected people have symptoms of varying degrees that range from mild to severe, including cough, fever, lethargy, anorexia, and muscle pain (8, 9). Besides, the temporary loss of smell and taste are also common symptoms in patients with COVID-19 (10). Researchers worldwide are using various techniques to develop drugs against SARS-CoV-2 infections, such as Remdesivir and Lopinavir, which previously have been chosen for human clinical trials (11, 12). In addition, several different types of COVID-19 vaccines have been approved globally to battle with COVID-19. However, the number of patients with COVID-19 is still rising rapidly (13, 14).

The World Health Organization considers epidemic hemorrhagic fever, Ebola virus Disease, influenza, SARS-CoV-2, SARS, and other diseases with epidemic or epidemic potential impacting human society substantially (15–17). The Hantavirus-caused epidemic hemorrhagic fever significantly influences public health, which primarily affected Asia and Europe. The incubation period for epidemic hemorrhagic fever is around 14-21 days. Headache and fever are the most common symptoms, often accompanied by lack of appetite, bleeding tendency, and renal failure (18). Fever, hypotension, oliguria, diuresis, and recovery are the five main clinical stages of epidemic hemorrhagic fever(EHF). Recently, both EHF and COVID-19 have outbroken simultaneously in some regions. It warns the possibility of co-infection of EHF and COVID-19. Thus, it also drives us to consider the strategies to treat this potential co-infection in advance. Although the type of pathogen and pathophysiology vary, several studies have shown that thrombosis in COVID-19 is associated with hemorrhage in viral hemorrhagic fever. Since the thrombogenic phase always precedes the bleeding phase, it can be considered a sequential change in the timing of acute infectious events (19). The two infections share similar symptoms and laboratory features early, posing challenges for accurate diagnosis and treatment. Due to the globalization of the economy and public transportation, many diseases have exceeded the limit of original geographic origins and are now affecting people worldwide (20). Previously, COVID-19 and dengue co-infection had placed a heavy burden on healthcare systems in dengue-endemic areas (21, 22). Therefore, to prevent the linked attacks of Hantavirus and COVID-19, we need more studies into treatments for these transmitted diseases.

**Figure 1:**
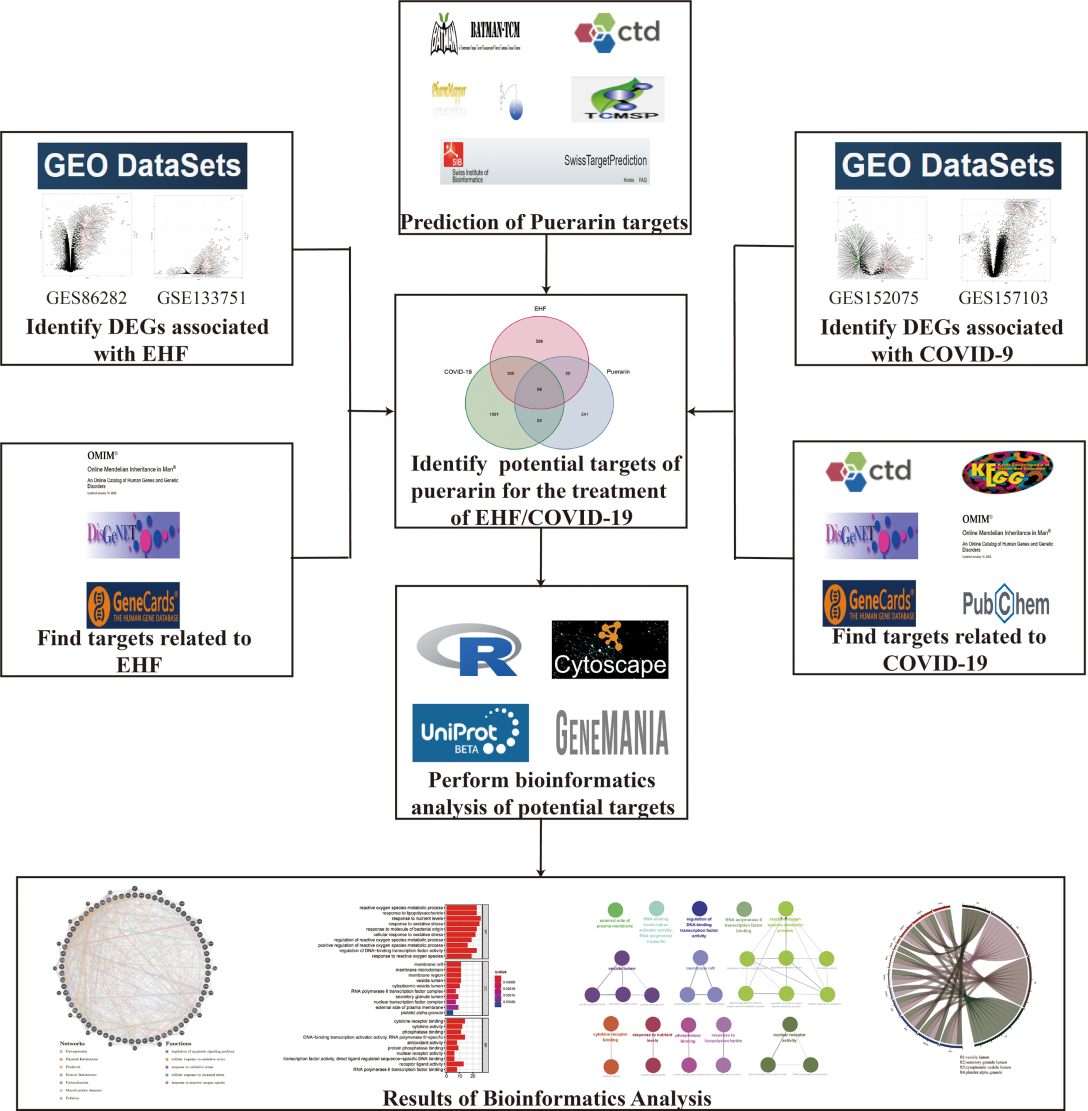
Flowchart. This figure shows the specific process to explore puerarin’s potential therapeutic effects and mechanisms in treating EHF/COVID-19 through network pharmacology and bioinformatics.

Puerarin is the main biologically active ingredient extracted from Pueraria Lobata, widely used in the therapy of various diseases such as diabetes, Parkinson’s disease, Alzheimer’s disease, and cancer (23). Studies have shown that puerarin may play important roles by regulating different pathways, including oxidative stress, cell cycle, inflammation, and autophagy (24). Puerarin may treat EHF/COVID-19 by modulating the immune system to enhance the induction of Tregs and modulate immune tolerance (25, 26). In conclusion, puerarin has potential therapeutic value in treating viral infections. Therefore, as shown in **Figure 1**, we analyzed genes regulated by puerarin, EHF, and COVID-19, through bioinformatic methods and obtained common core targets and common key pathways to explore whether puerarin could be a potential drug candidate for EHF/COVID-19.

## Materials and Methods

### Puerarin-Related Targets

We used PharmMapper (http://www.lilab-ecust.cn/pharmmapper/), Traditional Chinese Medicine Systems Pharmacology Database and Analysis Platform (TCMSP, https://tcmspw.com/tcmsp.php), comparative toxicogenomics database (CTD, http://ctdbase.org/), batman (http://bionet.ncpsb.org.cn/batman-tcm/index.php), and SwissTargetPrediction (http://www.swisstargetprediction.ch/) database to search for potential therapeutic targets of puerarin. Only the top 100 genes in each database were filtered for further analysis to increase the study’s accuracy. As the targets in the CTD database had no index for ranking, they were all included in present study.

### EHF/COVID-19-Associated Genes

The GSE157103, GSE152075, GSE86282, and GSE133751 datasets were downloaded from the gene expression omnibus website (https://www.ncbi.nlm.nih.gov/geo/) to collect EHF/COVID-19-related genes. DEGs were identified by using the “limma” package for R with a false discovery rate (FDR) <0.05 and log fold change (FC) absolute value > 1 as filter requirements. In addition, CTD (http://ctdbase.org/), Kyoto Encyclopedia of Genes and Genomes (KEGG, https://www.kegg.jp/), PubChem (https://pubchem.ncbi.nlm.nih.gov/), GeneCards (https://www.genecards.org/), OMIM (https://omim.org/), and DisGeNET (https://www.disgenet.org/) databases were also used to find genes associated with EHF and COVID-19.

### Construction of PPI Network

The “Venn” package for R was employed to identify common genes for puerarin, COVID-19, and EHF. The GeneMANIA database, a widely used bioinformatics database, could predict gene functions and build protein interaction networks in nine species, including humans. The obtained common genes were uploaded to the GeneMANIA database (http://genemania.org/) to develop the PPI network. To determine the key genes of puerarin in the therapy of EHF/COVID-19, we used the Cytoscape software to import the gene interaction network obtained from the GeneMANIA database and the cytohubba plug-in to compute and visualize the degree value of each gene.

### GO Term and KEGG Pathway Enrichment Analysis

Firstly, gene names were transformed into gene IDs through the UniProt website. Then, we carried out enrichment analysis of Gene Ontology (GO) and KEGG pathway through R language and visualized the results (only the top 30 results with q-value were displayed). Next, by using the ClueGo plug-in of the Cytoscape software, we conducted the correlation analysis on the findings of the enrichment analysis (only the results with the top 30 q-values were analyzed). Finally, the “circlize” package for R was used to display the highly correlated pathways and corresponding common genes.

### Analysis of Upstream Pathway Activity

The ability to distinguish the molecular origin of aberrant transcriptome regulation relied heavily on signaling pathway activity based on transcriptome data. By combining existing experimental data, the SPPED database could systematically obtain the consensus features of genes. It could predict differential expression patterns and upstream gene pathway activity more accurately than the usual collection of consensus features from a single data set. The SPEED2 database, which was utilized in this work, was based on SPEED and has been enhanced in several ways: (1) the number of anticipated pathways had been expanded to 16, (2) the gene dataset used to generate the database had been extended to 640, (3) the user was able to obtain upstream and downstream of the input gene list, and (4) statistical scores were provided to improve the accuracy of the prediction.

## Results

### Identification of Puerarin Targets

To obtain the relevant targets of puerarin, we selected 100, 125, 100, 54, and 96 targets from batman, CTD, PharmMapper, TCMSP, and SwissTargetPrediction database, respectively (**Supplementary Table 1**). After duplicate removal, a total of 415 targets were obtained.

### Identification of EHF/COVID-19-Associated Genes and Intersection With Puerarin

**Figure 2:**
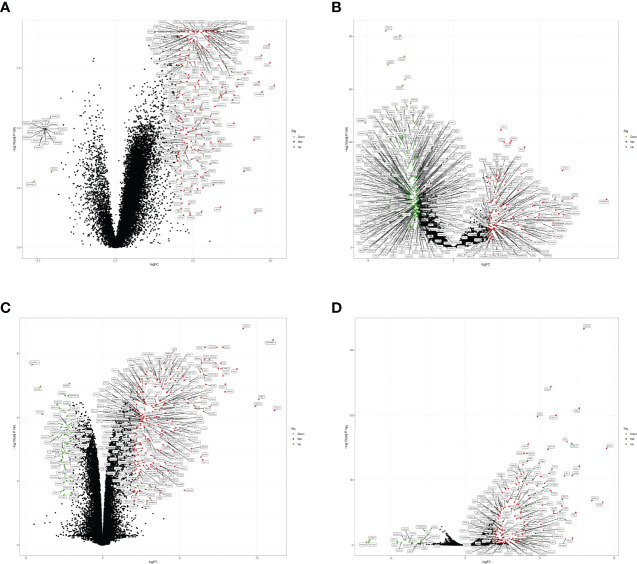
DEGs of COVID-19 and EHF. **(A)** Volcano plot of DEGs from the GSE157103 dataset. **(B)** Volcano plot of the DEGs from the GSE152075 dataset. **(C)** Volcano plot of DEGs from the GSE86282 dataset. **(D)** Volcano plot of the DEGs from the GSE133751 dataset. Red dots represented up-regulated genes in COVID-19 or EHF patients, and green dots represented down-regulated genes in COVID-19 or EHF patients (FDR<0.05 and absolute value of log(FC) > 2.

The GSE157103 dataset included in present study contained sequencing data of plasma samples from 100 COVID-19 positive patients and 26 normal individuals, while the GSE152075 dataset included sequencing data of nasopharyngeal swabs from 430 COVID-19 positive patients and 54 normal individuals. After analysis by the “limma” package for R, 444 and 245 DEGs were obtained, respectively (**Figures 2A, B**; **Supplementary Table 2**). 500/9865, 232/232, 500/655, 500/3329, 2/2 genes (**Supplementary Table 2**) were found related to COVID-19 through CTD, KEGG, PubChem, GeneCards and OMIM respectively. After combining the obtained genes and removing duplicate values, we attained 2260 genes related to COVID-19. We obtained 320 DEGs with GES86282 and 176 DEGswith GSE133751 datasets, respectively (**Figures 2C, D**; **Supplementary Table 3**).

Furthermore, based on OMIM, GeneCards, and DisGeNET databases, 109/109, 500/653, 51/51 genes (**Supplementary Table 3**) related to EHF were obtained. After removing duplication, 982 EHF-related genes were finally obtained.

**Figure 3:**
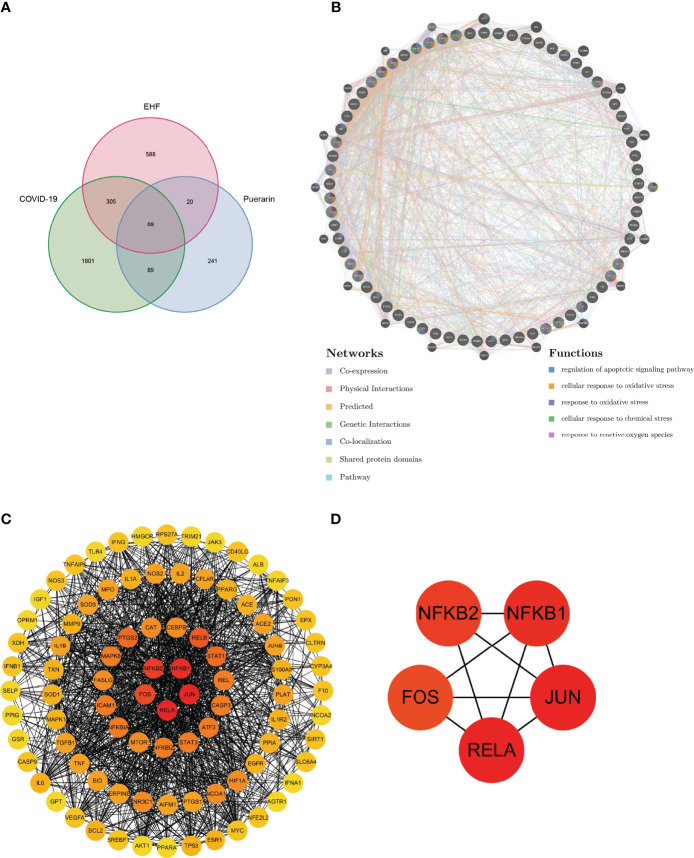
Analysis of common genes for COVID-19, EHF, and puerarin. **(A)** Venn diagram of the common gene. **(B)** PPI network of the common gene. **(C)** Degree value of each node of the PPI network. **(D)** The top five nodes in the PPI network degree value.

Then, we used the Venn diagram (**Figure 3A**) to show the intersection of COVID-19-related genes, EHF-related genes, and puerarin targets. 69 common genes (**Supplementary Table 4**) were obtained for subsequent bioinformatics analysis.

### Construction of PPI Network

We used the 69 common genes of COVID-19, EHF, and puerarin obtained in the previous steps to construct the Protein-Protein Interaction(PPI) network (**Supplementary Table 5**). The GeneMANIA database, based on multiple transcriptomic and proteomic databases such as GEO, BioGRID, and IRefIndex, had collected hundreds of millions of data sets and interactions and can be used to discover genes with similar functions and build interaction networks. We uploaded the common genes to the GeneMANIA database for PPI network construction. As shown in **Figure 3B**, we used different colored lines to show the relationship between genes, including Co-expression, Co-localization, Pathway, and Genetic Interactions. As shown in the bottom right of **Figure 3B**, different colors on gene nodes represented different functions (only the 5 functions with the smallest FDR values were shown). The molecular mechanism of puerarin in the therapy of EHF/COVID-19 might be related to oxidative stress and apoptosis. The degree value of the node was proportional to redness (**Figure 3C**). The five genes with the greatest degree values were RELA, JUN, NF-κB1, NF-κB2, and FOS (**Figure 3D**), suggesting that these genes might be important targets of puerarin in the therapy of EHF/COVID-19.

### Annotation of Common Genes

**Figure 4:**
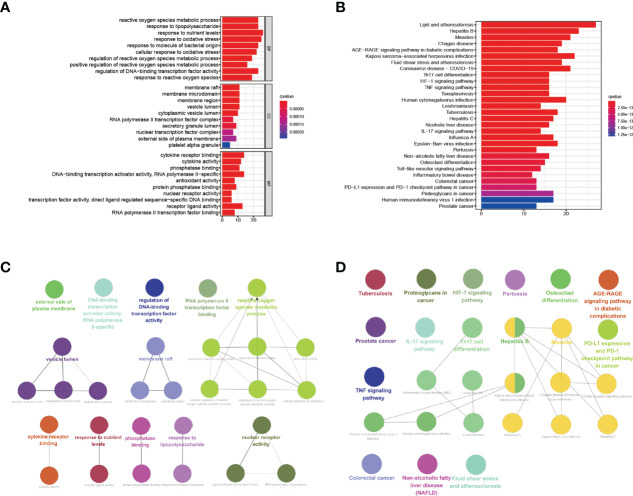
GO term and KEGG pathway enrichment analysis of puerarin, COVID-19, and EHF common genes (only the top 30 results). **(A, C)** exhibited the GO term and KEGG pathway enrichment analysis results of common genes, respectively. **(B, D)** exhibited the correlation analysis of the GO term and KEGG pathway enrichment analysis results of common genes, respectively.

We used bioinformatics analysis on 69 common genes to discover puerarin’s potential therapeutic value and mechanism on EHF/COVID-19. By Using the “clusterProfiler” package for R, we performed enrichment analysis on GO terms and KEGG pathways (**Figure 4**; **Supplementary Tables 6**, **7**).

The results of GO term enrichment analysis are displayed in **Figure 4A**. The GO biological process analysis found that the top ten processes were reactive oxygen species metabolic process, response to nutrient levels, and response to oxidative stress. There were four biological processes involved in oxygen metabolism, and it was hypothesized that puerarin could treat EHF/COVID-19 by influencing these processes.KEGG pathway enrichment analysis of 69 common genes showed that puerarin affects a series of signaling pathways, including coronavirus disease - COVID-19, Th17 cell differentiation, HIF-1 signaling pathway, TNF signaling pathway, Human cytomegalovirus infection, Tuberculosis, Influenza A, Epstein-Barr virus infection Pertussis, Toll-like receptor signaling pathway, and Human immunodeficiency virus 1 infection (**Figure 4B**).

**Figure 5:**
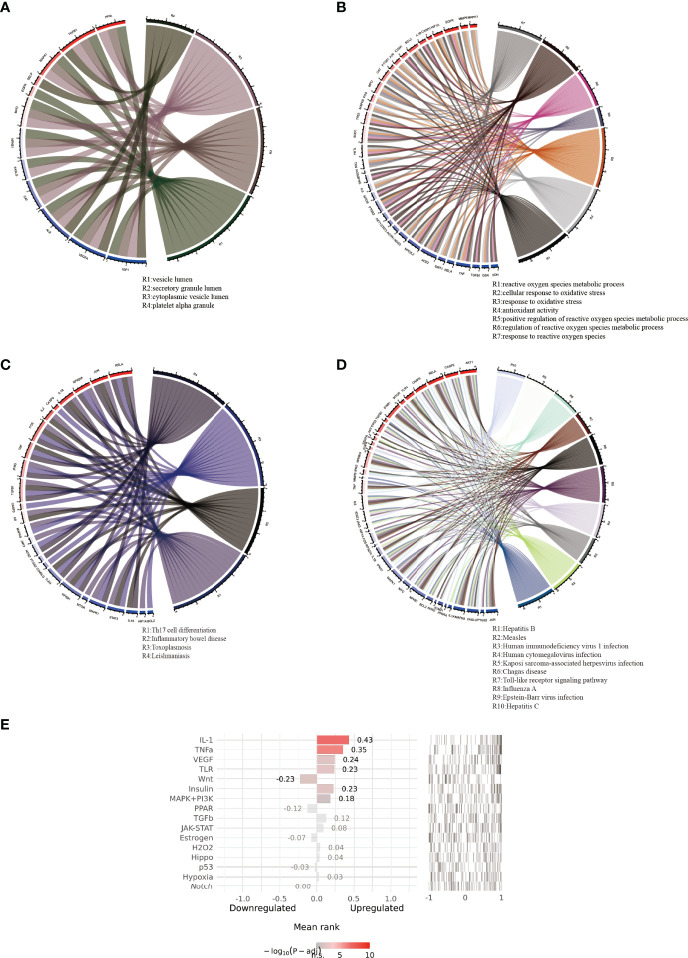
Pathways with high correlation and corresponding common genes. **(A, B)** respectively displayed the highly correlated pathways and corresponding common genes of GO term rich base results. **(C, D)** displayed the highly correlated pathways and corresponding common genes of KEGG pathway enrichment results. **(E)** displayed the results of upstream pathway activity analysis for 69 common genes.

By using the ClueGo plug-in of Cytoscape software, correlation analysis was performed on the top ten results of biological process, molecular function, and cellular component enrichment analysis findings in GO term and the top 30 pathways in term KEGG pathway enrichment analysis results. The GO term correlation analysis produced two significant groups (more than four connected nodes), as shown in **Figure 4C**. The largest categories was closely related to oxygen, including cellular response to oxidative stress, antioxidant activity, positive regulation of reactive oxygen species metabolic process, response to reactive oxygen species. The relationship between two categories of phrases and corresponding common genes was shown in **Figures 5A, B**. The results of the correlation analysis of the KEGG pathway are shown in **Figure 4D**. The largest category was closely related to viruses, including Hepatitis B, Measles, Human immunodeficiency virus 1 infection, Human cytomegalovirus infection. The relationship between two categories of phrases and corresponding common genes was shown in **Figures 5A–D**.

### Prediction of Upstream Pathway Activity

To further probe the probable molecular mechanism of puerarin in the therapy of EHF/COVID-19, the SPEED2 database was used to acquire the upstream pathway activities of common genes. The 16 upstream pathways were sorted according to adjusted p-values in **Figure 5C**. The smaller the corrected p-value is, the more significant the pathway is. As a result, puerarin’s mechanism of action in the therapy of EHF/COVID-19 could be associated with the IL-1, TNFα, and VEGF signaling pathways.

## Discussion

COVID-19, a rapidly expanding disease, is still spreading over the world. However, specific treatments for COVID-19 are still in development. COVID-19 has caused economic, social, health, and personal lifestyle difficulties across the globe. According to the most recent figures issued by Johns Hopkins University in the United States on January 7, 2022, the number of confirmed cases of COVID-19 worldwide has surpassed 300 million. While it takes only five months for the total number of infected people to reach to 300 million from 200 millions. As a global emerging zoonotic pathogen, Hantavirus causes EHF in Asia and Europe. Each year, more than 20,000 hantavirus cases are reported worldwide, most of which come from Asia (27). The number of Hantavirus cases has rapidly increased in recent years, posing a major threat to human health. EHF patients can also be infected with COVID-19; however, no effective anti-hantavirus/COVID-19 medications are available. The combination of traditional Chinese and Western medicine therapy modalities has been widely employed in the clinical therapy of patients with COVID-19. Traditional Chinese medicine can decrease the risk of severe disease of COVID-19 positive patients (28). The potential therapeutic value and molecular mechanism of puerarin in the therapy of EHF/COVID-19 were investigated. We found 69 possible therapeutic targets and molecular mechanisms by using bioinformatics for puerarin’s role in COVID-19 and EHF.

Various pharmacological studies against the SARS-CoV-2 and the host are currently being carried out. The narrow antiviral spectrum of antiviral drugs and the rapid evolution of drug resistance have led to a limited therapeutic effect on patients with COVID-19. Natural products are a key source for antiviral drug research and development, and puerarin has exhibited antiviral properties as a natural product. Studies showed that the mechanism of puerarin in inhibiting HIV-1 replication might be connected to limiting viral particles’ initial attachment to the cell surface of T lymphocytes and macrophages (29). Recent studies have shown that puerarin has antiviral effects and can inhibit neuraminidase *in vivo* and vitro (30). In addition, puerarin is a potential drug for treating Staphylococcus aureus-mediated infections, preventing Staphylococcus aureus from damaging human alveolar epithelial cells (31).

The common targets of COVID-19, EHF, and puerarin, according to present study, are ACE, ACE2, AGTR1, AKT1, BCL2, CASP3, CASP9, EGFR, ESR1, F10, GPT, and others. We hypothesize that RELA, JUN, NF-κB1, NF-κB2, and FOS are the main targets of puerarin in the therapy of EHF/COVID-19 based on protein interactions. Most patients with severe COVID-19 have elevated inflammatory cytokines and infection-related markers. Jun, FOS, or ATF, which bind to a shared AP-1 binding site, are subunits of the dimeric transcription factor AP-1. AP-1 factors can regulate different target genes to execute a range of biological tasks. c-Jun is involved in multiple cellular biological processes, including inflammatory responses. c-Jun can mediate the human inflammatory response induced by cytokines, thereby establishing a feedback loop to enhance the inflammatory effect after viral infection (32, 33). Therefore, drugs targeting c-Jun protein can be used to treat inflammatory diseases. FOS participates in human development by modifying the structure of cells as a stress response gene. The FOS induced by bacteria can activate the downstream immune cascade to eliminate bacteria (34).In mammals, NF-κB is widely expressed and regulates multiple cellular processes, including immunity, stress responses, and apoptosis. Viruses frequently target the NF-κB pathway to improve viral replication, host cell survival, and immune evasion. The NF-κB family has five members in human cells, including NF-κB1 and NF-κB2. By regulating numerous pro-inflammatory cytokines, chemokines, and adhesion molecules, NF-κB plays a role in the activation and recruitment of inflammatory regulatory cells (35). TNF and IL-1 can also influence NF-κB activation by strengthening the immune response and lengthening the time spent in the human body (36). RELA (also known as p65) is a member of the NF-κB family of proteins. The development of many inflammatory disorders is dependent on the huge rise in active p65 and the subsequent transactivation of effector molecules (37). Although the induction and activation of p65 are usually transient, it can activate downstream target genes with various functions, influencing cell biological processes such as proliferation and apoptosis (38, 39). As a key target in the inflammatory response, new drugs have been developed targeting p65. In conclusion, the molecular mechanism of puerarin in the treatment of EHF/COVID-19 may be strongly related to immune and inflammatory responses.

The results of the GO term enrichment revealed that the process of oxidative stress and oxygen metabolism might be linked to puerarin’s therapeutic effect. The disulfide-thiol balance plays an important role in viral entry into host cells, and oxidative stress from free radicals can disrupt this balance (40). COVID-19 has been proven in recent research to cause excessive oxidative stress, which can cause respiratory illness and even death in patients (41). Antioxidant supplementation can help to keep the human immune system stable and reduce the danger of SARS-CoV-2 infection. According to the biopsy of intestinal mucosa from EHF patients, the rise in Ki67 and cell proliferation could be linked to local oxidative stress (42). Puerarin can increase the expression of Nrf2 and HO-1 in diabetic mice, reducing oxidative stress and delaying cataract progression (43).

According to KEGG pathway enrichment analysis and literature, the molecular mechanism of puerarin in the therapy of EHF/COVID-19 may be related to HIF-1, TNF, IL-17, and Toll-like receptor signaling pathway. HIF-1α induced monocyte metabolic disturbance in patients with COVID-19 directly suppresses T cell responses and reduces epithelial cell survival (44). Only the mixture of TNF-α and IFN-γ induces cell death characterized by inflammatory cell death PANoptosis in Patients with COVID-19. The specific mechanism is that TNF-α and IFN-γ jointly activate the JAK/STAT1 axis, which drives caspase-8/FADD-mediated PANoptosis (45). As a result, neutralizing drugs targeting TNF-α and IFN-γ may be used in the clinical therapy of patients with COVID-19. In EHF patients, Hantavirus can work together with TNF- to stimulate the synthesis of ERK1/2, causing proteinuria and renal failure (46). Puerarin prevents normal hepatocyte apoptosis in septic mice by inhibiting the production of TNF-, IL-6, and IL-1 (47). According to clinical research, the deterioration of EHF is generally followed by an increase in cytokines such as IL-10, IL-17, and IL-12p70 (48). Through the MyD88-independent signaling pathway, Toll-like receptor (TLR)4, a member of TLRs family, may perform an anti-infective role against Hantavirus in the host (49). TLR4-mediated inflammatory signaling molecules are upregulated in patients with COVID-19 compared to healthy individuals, suggesting that TLR4 signaling may be associated with pathological inflammation in patients with COVID-19 (50). Puerarin reduces nickel-induced oxidative stress and inflammatory reactions *via* modulating the TLR4/p38/CREB signaling pathway, according to research (51).

We identified 69 core COVID-19, EHF, and puerarin targets and signaling pathways, including NF-κB, IL-17, TNF, TLR, and HIF-1. It investigated puerarin’s possible molecular mechanism in treating EHF/COVID-19 and demonstrated multi-targeted characteristics in treating infections. However, there are some limitation in present study. All of the data came from databases, and there was no follow-up *in vivo* or *in vitro* trials to back up the conclusions.

## Conclusions

In conclusion, we found that puerarin may treat patients with EHF/COVID-19 through anti-inflammatory, immunomodulatory, and antiviral, and explored potential therapeutic targets and molecular mechanisms of puerarin through bioinformatics analysis. These findings have not been tested in humans, but may demonstrate the potential value of Puerarin in the treatment of EHF/COVID-19.

## Data Availability Statement

The datasets presented in this study can be found in online repositories. The names of the repository/repositories and accession number(s) can be found in the article/**Supplementary Material**.

## Author Contributions

CZ developed the concept of the project. The data was collected and evaluated by WL, XL, and HW with the help of KN, QM, and JH. All authors reviewed and discussed the results and contributed to the paper preparation. CZ, WL, XL, and HW wrote the manuscript. All authors have read and approved the manuscript.

## Funding

The study was supported by Shenzhen Key Laboratory Foundation (ZDSYS20200811143757022).

## Conflict of Interest

The authors declare that the research was conducted in the absence of any commercial or financial relationships that could be construed as a potential conflict of interest.

## Publisher’s Note

All claims expressed in this article are solely those of the authors and do not necessarily represent those of their affiliated organizations, or those of the publisher, the editors and the reviewers. Any product that may be evaluated in this article, or claim that may be made by its manufacturer, is not guaranteed or endorsed by the publisher.

## Supplementary Material

Supplementary Table 1 Related targets of puerarin.

Supplementary Table 2 Related targets of COVID-19.

Supplementary Table 3 Related targets of EHF.

Supplementary Table 4 Common gene of COVID-19, EHF, puerarin.

Supplementary Table 5 PPI network of common genes.

Supplementary Table 6 GO term enrichment analysis of common genes.

Supplementary Table 7 KEGG pathway analysis of common genes.

## Abbreviations

EHF, epidemic hemorrhagic fever; PPI, protein-protein interaction; SARS-CoV-2, severe acute respiratory syndrome coronavirus 2; TCMSP, Traditional Chinese Medicine Systems Pharmacology Database and Analysis Platform; CTD, comparative toxicogenomics database; FDR, false discovery rate; FC, fold change; KEGG, Kyoto Encyclopedia of Genes and Genomes; GO, Gene Ontology; TLR, Toll-like receptor.
